# Gestational Weight Gain and Fetal-Maternal Adiponectin, Leptin, and CRP: results of two birth cohorts studies

**DOI:** 10.1038/srep41847

**Published:** 2017-02-02

**Authors:** Chad A. Logan, Rebecca Bornemann, Wolfgang Koenig, Frank Reister, Viola Walter, Giamila Fantuzzi, Maria Weyermann, Hermann Brenner, Jon Genuneit, Dietrich Rothenbacher

**Affiliations:** 1 Institute of Epidemiology and Medical Biometry, Ulm University, Ulm, Germany; 2 Department of Internal Medicine II - Cardiology, University Medical Center Ulm, Germany; 3 Department of Gynecology and Obstetrics, University Medical Center Ulm, Germany; 4 Division of Clinical Epidemiology and Aging Research, German Cancer Research Center (DKFZ), Heidelberg, Germany; 5 Department of Kinesiology and Nutrition, University of Illinois at Chicago, Chicago, IL, USA; 6 Faculty of Health Care Krefeld, Niederrhein University of Applied Sciences, Krefeld, Germany

## Abstract

Gestational weight gain (GWG) is an important modifiable factor known to influence fetal outcomes including birth weight and adiposity. Unlike behaviors such as smoking and alcohol consumption, the effect of GWG throughout pregnancy on fetal development and other outcomes has not been extensively studied. The aim of this study was to investigate the relationship of GWG with endocrine factors such as adiponectin, leptin, and C-reactive protein which may be associated with inflammatory response, fetal growth, and adiposity later in life. Data were obtained from the Ulm Birth Cohort Study (UBCS) and the Ulm SPATZ Health Study, two methodologically similar birth cohort studies including newborns and their mothers recruited from 11/2000–11/2001 and 04/2012–05/2013. In the two included birth cohorts we consistently observed statistically significant positive associations between GWG beginning as early as the second trimester with fetal cord blood leptin and stronger association beginning as early as the first trimester with post-delivery maternal serum leptin. Total weight gain exceeding commonly accepted recommended guidelines was consistently associated with higher leptin levels in both cord blood and post-delivery maternal serum. These results suggest a potential pathomechanistic link between fetal environment and surrogate markers of long-term health.

Mounting evidence suggests fetal environment and nutrition may play a role in the origin of a number of chronic diseases manifesting later in life^1^. For example, numerous studies have reported associations between intrauterine growth restriction and cardio-metabolic health beginning in early childhood which may then persist throughout life contributing to higher risk for obesity, heart disease, and type 2 diabetes^2^. Though identification of specific mechanisms remains unresolved, it is widely thought that fetal growth may be mediated by nutritional availability during critical periods of gestation when organs and organ systems undergo periods of “plasticity” and are therefore subject to fetal programming by epigenetic modification^3^,^4^.

Though a number of confounding factors such as offspring gender, parity, and smoking are known to influence fetal growth^5^,^6^, fetal resource availability is primarily associated with maternal size and body composition, dietary intake, and placental function^7^,^8^. In relation to these factors, one large scale retrospective study comparing successive births within mothers identified overall gestational weight gain (GWG) as a contributing factor to birthweight^9^. Furthermore, strong association has been observed between trimester specific GWG and fetal ultrasound measures^10^. Yet, it remains unclear why differences in resource availability throughout the gestational period may contribute to or modify associations between fetal growth and long-term health.

One potential mechanism has been identified in studies observing associations between cord blood levels of the hormones adiponectin and leptin and pre- and post-natal growth. These hormones are involved in signaling pathways associated with growth, metabolism, and the immune system thus providing a possible link between fetal growth and disease^11^,^12^,^13^,^14^. Though adiponectin and leptin are normally both correlated with adiposity and negatively correlated with each other, leptin levels are also known to increase throughout pregnancy as the placenta becomes a major source of leptin synthesis^15^. Several studies have reported positive correlations between cord blood adiponectin and leptin levels and either birthweight or size for gestational age^16^,^17^,^18^, as well as, body mass index (BMI) in children up to 3 years of age^19^,^20^. Altogether, these results suggest fetally programmed adipokine levels may play a role as a potential biological mechanism for such associations, but require further refined analyses and corroboration in independent cohorts.

The primary aim of this analysis was to investigate associations between GWG throughout pregnancy and both fetal (cord blood) and maternal (serum collected at birth) levels of adiponectin and leptin. As we have previously identified potential associations between leptin and birth-related factors including duration of labor^21^, we also investigate associations with the acute inflammatory marker high sensitivity C-reactive protein (hs-CRP) which may be indicative of more proximal inflammatory response. All three biomarkers represent three factors which may be potentially associated with intrauterine growth and/or long-term cardio-metabolic health. In contrast to previous studies which have only reported associations observed with either cord blood or maternal serum levels of each biomarker, fetal and maternal outcomes were also directly compared in order to explore potential pathomechanistic associations.

## Methods

### Study design and population

Data were obtained from the Ulm Birth Cohort Study (UBCS) and the Ulm SPATZ Health Study, two methodologically similar birth cohort studies including newborns and their mothers recruited from the general population shortly after delivery in the University Medical Center Ulm, Southern Germany, respectively from 11/2000–11/2001 and 04/2012–05/2013. Details can be found elsewhere^22^,^23^.

Exclusion criteria were outpatient delivery, maternal age <18 years, transfer of the newborn or the mother to intensive care immediately after delivery, and/or insufficient knowledge of the German (UBCS and SPATZ), Turkish, or Russian (both UBCS only) language. At baseline, the UBCS and SPATZ cohorts respectively included 1090 newborns of 1066 mothers (67% of all 1593 eligible families) and 1006 newborns of 970 mothers (49% of all 1999 eligible families). For the purposes of this analysis, the study populations were restricted to singleton full-term (gestational age ≥37 weeks) newborns. In order to ensure comparability among results, we also restricted to mother-infant pairs for whom results were obtained for all three biomarkers in both cord blood and post-delivery maternal serum. Ethical approval was obtained from and all study protocols were carried out in accordance with guidelines approved by the ethics board of Ulm University (UBCS: no. 98/2000, SPATZ: no. 311/11). Participation was voluntary and written informed consent was obtained in each case. Exposure, outcome, and confounder definitions as well as statistical methods were identical for both studies unless specifically stated otherwise.

### Data collection

Maternal demographic and lifestyle data including age at delivery, education (<12 years or ≥12 years), parity (first birth or greater), smoking history (within the year prior to pregnancy) were collected using a self-administered questionnaire during the hospital stay following delivery. Clinical data related to the child’s delivery including gender, birthweight, delivery mode, and duration of labor were obtained from electronic hospital records. Clinical data related to the mother’s pregnancy, including gestational weight measurements, were obtained from routine paper documentation known in Germany as “Mutterpass”, which obstetricians are required to issue to their patients when pregnancy is clinically established and which are generally updated at each clinical visit during pregnancy.

### Gestational weight gain (GWG)

Pre-pregnancy weight and height were estimated based on self-reported data documented in “Mutterpass” during the mother’s first obstetric appointment at which pregnancy was clinically established. Trimester specific and total weight gain were calculated using obstetrician-documented weight measured nearest to the end of the first (day 85), second (day 190), and third (day closest to full-term delivery) trimesters. Start of pregnancy was calculated as 280 days before the obstetrician reported full-term delivery date estimated at approximately 12 weeks gestation using fetal ultrasound measurements. As weights were seldom recorded on the trimester end-date, only weights recorded on the closest day within 14 days of the target date were used in the analysis^24^. In order to account for this difference and those associated with body type, weights were converted into z-scores obtained from linear regression models of the effect of gestational age on GWG adjusted for pre-pregnancy BMI. To improve interpretability of model results, total GWG was also analyzed as the difference from mean recommended GWG by BMI category as specified by the Institute of Medicine (IOM) [BMI category = mean recommended weight gain: Underweight (BMI < 18.5) = 15.45+/−2.73 kg; Normal (18.5 ≤ BMI < 25.0) = 13.64+/−2.27 kg; Overweight (25.0 ≤ BMI < 30.0) = 9.09+/−2.27 kg; Obese (BMI ≥ 30.0) = 7.05+/−2.05 kg]^25^.

### Adiponectin, leptin, and hs-CRP

Cord blood was collected in S-Monovette 7.5 ml serum-gel tubes (Sarstedt AG & Co, Nümbrecht, Germany) by midwives or obstetricians shortly after delivery, centrifuged, and stored in a refrigerator until further processed for long-term storage at −80 °C by trained study personnel. In SPATZ, average time until long-term storage was 2.4 days (sd = 0.8 days); in UBCS the process was similar, but the time was not recorded. Maternal blood was collected during routine blood collection following delivery (after 24 h for most mothers). Adiponectin, leptin (both R&D Systems GmbH, Wiesbaden, Germany), and high-sensitivity CRP (hs-CRP, Immunodiagnostik AG, Bensheim, Germany) concentrations were measured by ELISA (SPATZ: n = 837; UBCS: n = 900). Biomarker measurements reported as below detection limit (SPATZ: n = 1) were imputed to the lower detection limit. Hs-CRP levels above 200 μg/L have been associated with amniotic fluid infection, therefore subjects with cord blood hs-CRP measurements above 200 μg/L were excluded from the analysis^26^.

For UBCS, adiponectin and leptin were measured in 06-07/2005 in Heidelberg, Germany, whereas hs-CRP was measured in 08-10/2009 in a research lab at Medical Center Ulm from continuously frozen stored material. For SPATZ, all three markers were measured in the latter research lab in cord blood and post-delivery maternal serum in 06-08/2013 with the same equipment and by the same technician as in UBCS. Hs-CRP levels differed between both studies with the whole distribution shifted by approximately 10 μg/L towards higher levels in UBCS^21^. With identical lab and technician as well as similar standard curves and controls in both studies we attribute this to the longer duration of long-term storage in UBCS (up to 9 years vs. up to 1.25 years in SPATZ).

### Statistical analyses

To assess the impact of missing data and restrictions, characteristics (proportions or means) of each study sample were compared to 95% confidence intervals of the characteristics within the corresponding full cohort. Chi-square and Kruskall-Wallis tests were performed to identify significant differences (alpha = 0.05) in characteristics across study cohorts. Biomarkers were log transformed to account for right skewed distributions (leptin and CRP) or for comparability (adiponectin). Biomarker measurements outside 3 standard deviations from the underlying mean were considered potential outliers and excluded in sensitivity analyses. Linear regression models were used to estimate geometric means ratios (GMR) for the effect of a one standard deviation difference from the underlying mean trimester specific and total GWG and of a 5 kg change in difference from IOM recommended weight gain separately with each biomarker (cord blood and post-delivery maternal serum). Maternal age, education, parity, smoking history, and pre-pregnancy BMI, as well as, the child’s gender, birthweight, mode of delivery, and duration of labor were tested as putative confounding factors. As crude effects were small, putative confounders producing a 1% change in GMR after single adjustment of models measuring the effect of total GWG on any cord blood biomarker were selected for inclusion in adjusted models. All statistical analyses were performed by using SAS 9.4 (SAS Institute, Cary, NC).

## Results

Following restriction to singleton full-term newborns, complete GWG and valid biomarker data were available for 540 UBCS and 412 SPATZ subjects (Supplement Figure 1). Due to the restriction to singleton full-term births, mean birthweights and proportions of vaginal spontaneous delivery in the analysis datasets were slightly higher than those observed in their respective cohort populations (Supplement Tables 1 and 2). Compared to UBCS, higher maternal age and higher proportions of mothers with higher education, no recent history of smoking, and elective cesarean or vaginally assisted birth were observed in SPATZ (Table 1). Approximately 50% of mothers in both cohorts were classified as having experienced “excessive” weight gain above the IOM recommendation limit for their respective pre-pregnancy BMI status by the end of pregnancy.

**Figure 1:**
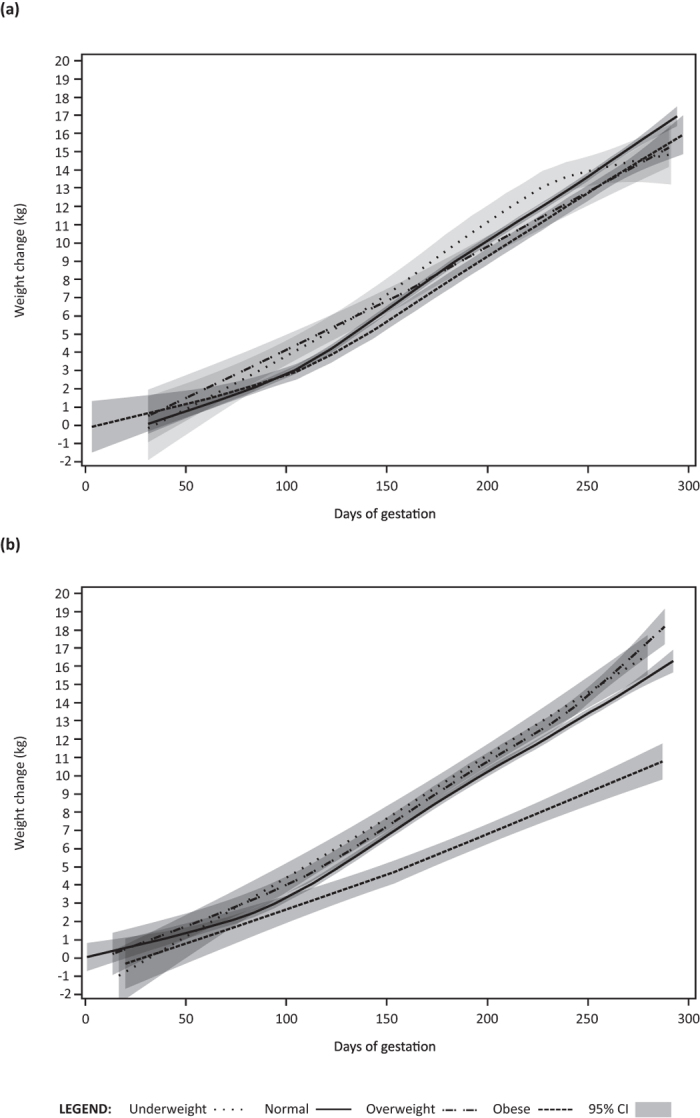
UBCS (**a**) and SPATZ (**b**) gestational weight gain trajectories by BMI category.

Overall, GWG trajectories were comparable between cohorts with the exception of obese SPATZ mothers who appeared to exhibit a lower weight gain trajectory following the first trimester (Fig. 1). By delivery, total weight gained by obese mothers was significantly lower in SPATZ (mean: 10.6 kg, SD: 8.0) than in UBCS (mean: 15.0 kg, SD: 7.6; p = 0.02) and less likely to be classified as excessive (p = 0.02). The difference was likely due to skewed distribution toward higher BMI among obese mothers in the SPATZ (40% of subjects ≥35 kg/m^2^) cohort compared to that observed in UBCS (16% of subjects).

In our models we observed consistent positive crude association between GWG z-score and cord blood leptin beginning as early as the second trimester (data not shown). Following adjustment for maternal smoking history and duration of labor, associations were slightly attenuated but remained significant (p < 0.05) in UBCS and for the 3^rd^ trimester in SPATZ (Table 2). Patterns of association with cord blood hs-CRP resembled those for cord blood leptin in UBCS and beginning in the 3^rd^ trimester in SPATZ. Following additional adjustment for gestational age and BMI, increasing GWG relative to IOM recommendations showed patterns of association similar to those observed for standardized total weight gain. Similar but stronger patterns of association were observed between all GWG measures and leptin measured in post-delivery maternal serum in both cohorts and beginning as early as the 1^st^ trimester in UBCS (Table 3). No association was observed in either study between GWG measures and adiponectin in post-delivery maternal serum in UBCS and after adjustment in SPATZ. Point estimates did not significantly change following exclusion of outlying biomarker measurements.

**Figure 2:**
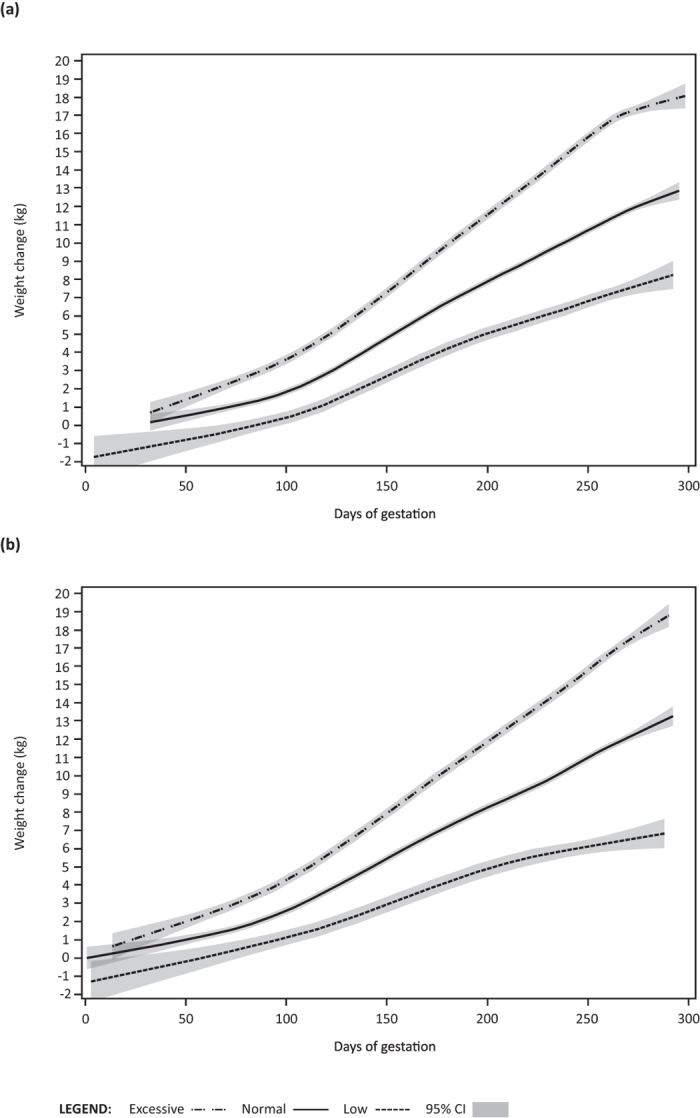
UBCS (**a**) and SPATZ (**b**) gestational weight gain trajectories by IOM category.

To further explore strong associations observed between difference from IOM-recommended weight gain and leptin, we plotted weight gain trajectories for subjects who were categorized as below (“low”), within (“normal”), or above (“excessive”) specified recommended total weight gain ranges (Fig. 2)^25^. In both cohorts, mothers classified as having had “excessive” total weight gain exhibited higher rates of weight gain beginning by the 7^th^ week of pregnancy compared to those classified as having “low” or “normal total pregnancy weight gain.

## Discussion

In our analyses of two birth cohorts recruited from the general population with identical sampling and measurement methodologies and a time lag of 11 years, we observed significant positive association between GWG beginning as early as the second trimester and cord blood leptin. GWG beginning as early as the first trimester was associated with higher levels of post-delivery maternal serum leptin measured shortly after birth. Total weight gain exceeding IOM recommendations was consistently associated with higher leptin levels in both cord and post-delivery maternal serum. Finally, first trimester GWG was indicative of IOM total weight gain category. These results suggest GWG beginning as early as the first trimester might influence expression of leptin in the fetus, the newborn and possibly later in life. Furthermore, lack of consistent association between GWG and hs-CRP suggests the association with leptin was not likely associated with the inflammatory response at birth.

Though we identified two previous studies reporting similar findings with cord blood outcomes, we believe we are among the first to concurrently report on associations between total and trimester-specific GWG and adiponectin, leptin, and hs-CRP levels in both cord blood and post-delivery maternal serum. Our results support those of the Rhea group, who have previously identified similar positive associations between IOM-recommended GWG and cord blood leptin^27^,^28^. Furthermore, our results support the lack of association for the effect of GWG on cord blood adiponectin levels^29^. Lastly, our results also correlate with a recent study reporting a positive association between levels of post-delivery maternal serum leptin during the second trimester and subsequent GWG^30^.

Interestingly, we observed similar but stronger patterns of association between GWG and leptin levels measured in post-delivery maternal serum compared to those observed in cord blood. Maternal serum leptin increases beginning in early pregnancy before peaking in the 3^rd^ trimester^31^. As maternal serum leptin levels decrease sharply after delivery, it is likely that increases observed during pregnancy are primarily the result of release of placental leptin rather than from adipose tissue^32^. In contrast, cord blood leptin levels have been previously associated with birthweight and neonatal adiposity, suggesting the newborn’s adipocytes as the most likely source of cord blood leptin^16^,^21^. Strong correlation observed in our data between cord blood leptin, but not post-delivery maternal serum leptin, and birth weight in both UBCS and SPATZ further supports this hypothesis (Supplement Table 3).

Though we observed correlation between fetal and maternal leptin levels in our cohorts (Supplement Table 3), it is currently unclear whether exchange of leptin occurs across the placenta during pregnancy. At least one *in-vitro* study has demonstrated the possibility of cross-placental transfer of circulating leptin in human choriocarcinoma BeWo cells^33^. Furthermore, at least a small percentage of placental leptin is thought to be secreted into fetal circulation^32^. Though it is possible fetal leptin levels could be influenced by leptin originating from the placenta and/or maternal adipose tissue which may ultimately affect fetal growth, maternal circulating leptin is not directly associated with birthweight^31^. Therefore, despite observable correlation between maternal and fetal leptin levels, it remains unclear whether associations between GWG and post-delivery maternal serum leptin may be directly or indirectly indicative of the weaker associations observed in cord blood.

Maternal leptin levels in pregnancy have also been associated with pregnancy-related health conditions including gestational diabetes and pre-eclampsia^34^. Though complete data was available for UBCS, little change in fetal or maternal biomarker point estimates after exclusion of SPATZ mothers with diagnosed (n = 44) or suspected (n = 4) gestational diabetes. However, as biomarkers were only measured upon delivery, it remains unknown whether stronger associations would have been observed earlier in pregnancy before treatment regimens (eg. insulin or dietary changes) could be established.

The key strength of our study was our ability to replicate results in two geographically and methodologically similar but demographically different cohorts. Despite the demographic differences, GWG, biomarker outcomes, and overall results were similar to one another. However, it should be noted that point estimates for both cord blood and post-delivery maternal serum leptin were consistently higher in the UBCS cohort. Though we were unable to identify a specific reason for this difference, factors associated with differences between studies in GWG trajectory amongst obese mothers may warrant further exploration.

As with other studies of this nature, some key limitations must also be acknowledged. First, approximately 31% of eligible UBCS mothers and 43% of eligible SPATZ mothers were excluded from our analyses due primarily to restriction to mothers with gestational weight measurements reported within 14 days of the end of each trimester and delivery. In most cases, these subjects were missing first trimester weight measurements which may have been due to a number of factors, including late establishment of pregnancy. However, no significant differences in demographic or lifestyle factors were observed due to missing data. Furthermore, several sensitivity analyses in which we included all available data for each model, less stringent restriction, and/or wider acceptable date ranges for trimester-specific weight gain estimation produced similar point estimates to reported results. Second, biomarkers were only measured upon (cord blood) and after (maternal serum) delivery and post-delivery maternal serum collection time varied. As GWG was only marginally associated with hs-CRP in UBCS cord blood and not associated with hs-CRP in maternal serum, it is unlikely more proximal factors at birth, such as duration of labor, affected levels of the other biomarkers. Nevertheless, we were unable to specifically determine precisely when and how biomarkers may have changed over the course of pregnancy or the possibility of reverse causality. Lastly, although specific timing of post-delivery maternal serum collection was unavailable in UBCS, we observed a weak but significant correlation between duration from delivery to blood collection and maternal adiponectin and leptin respectively in SPATZ. Adjustment for time to collection did not affect SPATZ post-delivery maternal serum model results.

Despite certain limitations, our results from two independent cohorts provide strong evidence supporting an association between GWG and fetal-maternal leptin. Although GWG is likely only one of a number of factors associated with leptin synthesis or expression, our results are in line with the idea that GWG may be an important exposure during early fetal development which may potentially influence long-term health in offspring. Therefore, further research may be warranted to prove and better understand causality of the association and the potential effects on fetal development, as well as the long-term consequences of hypo- and hyper-leptinemia during gestation.

## Additional Information

**How to cite this article:** Logan, C. A. *et al*. Gestational Weight Gain and Fetal-Maternal Adiponectin, Leptin, and CRP: results of two birth cohorts studies. *Sci. Rep.* **7**, 41847; doi: 10.1038/srep41847 (2017).

**Publisher's note:** Springer Nature remains neutral with regard to jurisdictional claims in published maps and institutional affiliations.

## Supplementary Material

Supplement Data

## Figures and Tables

**Table 1:** Comparison of maternal and birth related factors in the UBCS and SPATZ cohorts^†^.

	UBCS (n = 540)*		SPATZ (n = 412)*		p-value**
Maternal Factors					
Maternal age (years)	539	31.3 (27.1; 34.7)	412	32.5 (29.8; 36.0)	<0.001
Maternal education					<0.001
≥12 years education	192	(36.3%)	250	(61.7%)	
<12 years education	337	(63.7%)	155	(38.3%)	
Parity					0.270
First parity	276	(51.5%)	227	(55.1%)	
Second or higher	260	(48.5%)	185	(44.9%)	
Smoking history (within 1 year before delivery)					0.004
No	361	(66.9%)	307	(75.4%)	
Yes	179	(33.1%)	100	(24.6%)	
Maternal pre-pregnancy BMI category					0.057
Underweight (BMI < 18.5)	17	(3.2%)	12	(2.9%)	
Normal (18.5 ≤ BMI < 25.0)	364	(67.5%)	257	(62.7%)	
Overweight (25.0 ≤ BMI < 30.0)	120	(22.3%)	91	(22.2%)	
Obese (BMI ≥ 30.0)	38	(7.1%)	50	(12.2%)	
Pregnancy and Birth					
Gender					0.358
Male	280	(51.9%)	226	(54.9%)	
Female	260	(48.1%)	186	(45.1%)	
Birth weight (g)	540	3420 (3130; 3750)	412	3410 (3120; 3708)	0.733
Delivery mode					<0.001
Vaginal spontaneous	448	(83.0%)	274	(66.5%)	
Elective cesarean	23	(4.3%)	53	(12.9%)	
Emergency cesarean	48	(8.9%)	42	(10.2%)	
Vaginal assisted	21	(3.9%)	43	(10.4%)	
Duration of labor (hours)	533	6.9 (4.1; 11.9)	400	6.9 (3.4; 11.1)	0.102
Gestational age at Gestational Weight Measure (days)					
Beginning of trimester 2	540	85 (80; 90)	412	84 (78; 90)	0.527
Beginning of trimester 3	540	192 (185; 198)	412	189 (183; 196)	0.176
Last measure	540	275 (268; 281)	412	273 (267; 280)	0.045
Gestatational Weight Gain (kg)					
Trimester 1	540	1.6 (0.3; 3.4)	412	1.9 (0.5; 3.1)	0.719
Trimester 2	540	7.3 (5.6; 9.0)	412	7.0 (5.4; 8.6)	0.049
Trimester 3	540	5.7 (3.9; 7.7)	412	5.6 (3.8; 7.4)	0.510
Total	540	15.0 (11.8; 18.8)	412	14.5 (11.6; 18.0)	0.145
Weight Gain Category (IOM, 2009)					0.584
Low	85	(15.8%)	66	(16.1%)	
Normal	175	(32.5%)	145	(35.4%)	
Excessive	279	(51.8%)	199	(48.5%)	
Cord Blood Markers					
Adiponectin (mg/l)	540	31.1 (23.0; 40.5)	412	29.9 (21.9; 38.7)	0.250
Leptin (μg/l)	540	7.9 (4.8; 13.0)	412	7.4 (4.7; 12.3)	0.464
hs-CRP (μg/l)	540	39.8 (26.1; 63.5)	412	31.0 (20.4; 48.7)	<0.001
Post-Delivery Maternal Serum Markers					
Adiponectin (mg/l)	540	8.8 (6.1; 11.6)	412	5.7 (4.2; 7.9)	<0.001
Leptin (μg/l)	540	13.9 (7.1; 27.2)	412	13.3 (6.9; 25.8)	0.318
hs-CRP (mg/l)	540	53.9 (33.1; 80.9)	412	67.2 (34.7; 108.6)	<0.001

Abbreviations: UBCS = Ulm Birth Cohort Study; IOM = Institute of Medicine.

^†^Statistics expressed as n and column percentage for categorical variables or median (25^th^ percentile; 75^th^ percentile) for continuous variables.

*Column values may not always add up to total due to missing values for some variables.

**p-value calculated as either χ^2^ or fisher’s exact for categorical variables or Kruskal-Wallis for continuous variables.

**Table 2:** Adjusted associations of gestational weight gain with cord blood biomarkers.

	Adiponectin (mg/l)				Leptin (μg/l)				hs-CRP (μg/l)			
	UBCS		SPATZ		UBCS		SPATZ		UBCS		SPATZ	
	GMR	95%CI	GMR	95%CI	GMR	95%CI	GMR	95%CI	GMR	95%CI	GMR	95%CI
Gestational weight gain^a^												
1st trimester	1.03	[0.99, 1.08]	1.00	[0.95, 1.05]	1.00	[0.94, 1.08]	0.98	[0.91, 1.06]	1.01	[0.96, 1.06]	1.01	[0.95, 1.07]
2nd trimester	0.99	[0.95, 1.03]	1.06	[1.01, 1.12]*	1.11	[1.03, 1.19]**	1.05	[0.97, 1.14]	1.06	[1.01, 1.12]*	0.99	[0.93, 1.06]
3rd trimester	1.02	[0.98, 1.07]	1.03	[0.98, 1.08]	1.14	[1.06, 1.22]***	1.09	[1.01, 1.17]*	1.04	[0.99, 1.09]	1.06	[0.99, 1.13]
Total	1.02	[0.98, 1.07]	1.04	[0.99, 1.10]	1.13	[1.05, 1.21]***	1.07	[1.00, 1.16]	1.06	[1.00, 1.11]*	1.04	[0.98, 1.11]
Difference from IOM recommended weight gain (per 5 kg increase)^b^	1.02	[0.98, 1.06]	1.04	[0.99, 1.09]	1.11	[1.04, 1.18]***	1.08	[1.01, 1.15]*	1.05	[1.00, 1.10]*	1.04	[0.98, 1.09]

Abbreviations: UBCS = Ulm Birth Cohort Study; hs-CRP (high sensitivity C-reactive protein); IOM = Institute of Medicine.

UBCS subjects were recruited from 11/2000–11/2001; SPATZ subjects were recruited from 04/2012–05/2013.

GMR: geometric means ratio for a one standard deviation increase (GWG) or a 5 kg increase (IOM); 95%CI: 95% confidence interval.

^a^Adjusted for history of smoking and duration of labor.

^b^Adjusted for history of smoking, pre-pregnancy BMI, duration of labor, and gestational age at delivery.

^*^Asterisks indicate p-value significance (*) <0.05; (**) <0.01; (***) <0.001.

**Table 3:** Adjusted associations of gestational weight gain with post-delivery maternal serum biomarkers.

	Adiponectin (mg/l)				Leptin (μg/l)				hs-CRP (mg/l)			
	UBCS		SPATZ		UBCS		SPATZ		UBCS		SPATZ	
	GMR	95%CI	GMR	95%CI	GMR	95%CI	GMR	95%CI	GMR	95%CI	GMR	95%CI
Gestational weight gain^a^												
1st trimester	1.03	[0.99, 1.08]	1.00	[0.95, 1.04]	1.10	[1.01, 1.19]^*^	1.05	[0.96, 1.14]	0.97	[0.91, 1.03]	0.97	[0.89, 1.06]
2nd trimester	0.99	[0.95, 1.03]	0.98	[0.93, 1.02]	1.26	[1.16, 1.37]^***^	1.14	[1.05, 1.25]^**^	1.00	[0.94, 1.07]	1.05	[0.96, 1.15]
3rd trimester	1.02	[0.98, 1.07]	0.96	[0.92, 1.01]	1.47	[1.35, 1.59]^***^	1.30	[1.20, 1.41]^***^	1.07	[1.00, 1.13]^*^	1.04	[0.96, 1.13]
Total	1.02	[0.98, 1.07]	0.97	[0.92, 1.01]	1.42	[1.31, 1.54]^***^	1.29	[1.19, 1.40]^***^	1.02	[0.95, 1.08]	1.05	[0.96, 1.14]
Difference from IOM recommended weight gain (per 5 kg increase)^b^	1.02	[0.98, 1.06]	0.97	[0.93, 1.00]	1.37	[1.28, 1.47]^***^	1.29	[1.21, 1.37]^***^	1.01	[0.96, 1.07]	1.05	[0.98, 1.13]

Abbreviations: UBCS = Ulm Birth Cohort Study; hs-CRP (high sensitivity C-reactive protein); IOM = Institute of Medicine.

UBCS subjects were recruited from 11/2000–11/2001; SPATZ subjects were recruited from 04/2012–05/2013.

GMR: geometric means ratio for a one standard deviation increase (GWG) or a 5 kg increase (IOM); 95%CI: 95% confidence interval.

^a^Adjusted for history of smoking and duration of labor.

^b^Adjusted for history of smoking, pre-pregnancy BMI, duration of labor, and gestational age at delivery.

^*^Asterisks indicate p-value significance (*) <0.05; (**) <0.01; (***) <0.001.
