# Supplementary material for: Gestational Weight Gain and Fetal-Maternal Adiponectin, Leptin, and CRP: results of two birth cohorts studies
Source: Sci Rep. 2017 Feb 2;7:41847. doi: 10.1038/srep41847 (PMC5288774; doi:10.1038/srep41847)
Supplement: Supplement Data [file srep41847-s1.pdf]

Gestational Weight Gain and Fetal-Maternal Adiponectin, Leptin, and CRP: results of two birth cohorts studies

Chad A. Logan [1], Rebecca Bornemann [1], Wolfgang Koenig [2], Frank Reister [3], Viola Walter [4], Giamila Fantuzzi[5], Maria Weyermann[6], Hermann Brenner [4], Jon Genuneit [1,7], Dietrich Rothenbacher [1*]

Affiliations:

1 Institute of Epidemiology and Medical Biometry, Ulm University, Ulm, Germany

2 Department of Internal Medicine II - Cardiology, University Medical Center Ulm, Germany

3 Department of Gynecology and Obstetrics, University Medical Center Ulm, Germany

4 Division of Clinical Epidemiology and Aging Research, German Cancer Research Center (DKFZ), Heidelberg, Germany

5Department of Kinesiology and Nutrition, University of Illinois at Chicago, Chicago, IL, USA

6 Faculty of Health Care Krefeld, Niederrhein University of Applied Sciences, Krefeld, Germany

7 Member of ‘In-FLAME’ the International Inflammation Network, World Universities Network (WUN)

Address correspondence to: Prof. Dr. med. Dietrich Rothenbacher, MPH, Institute of Epidemiology and Medical Biometry, Ulm University, Helmholtzstr. 22, D-89081 Ulm, Germany [dietrich.rothenbacher@uni-ulm.de], phone: 0049 731 500 31060, fax: 0049 731 500 31069

Supplement figure 1: Flow chart of the study population restrictions

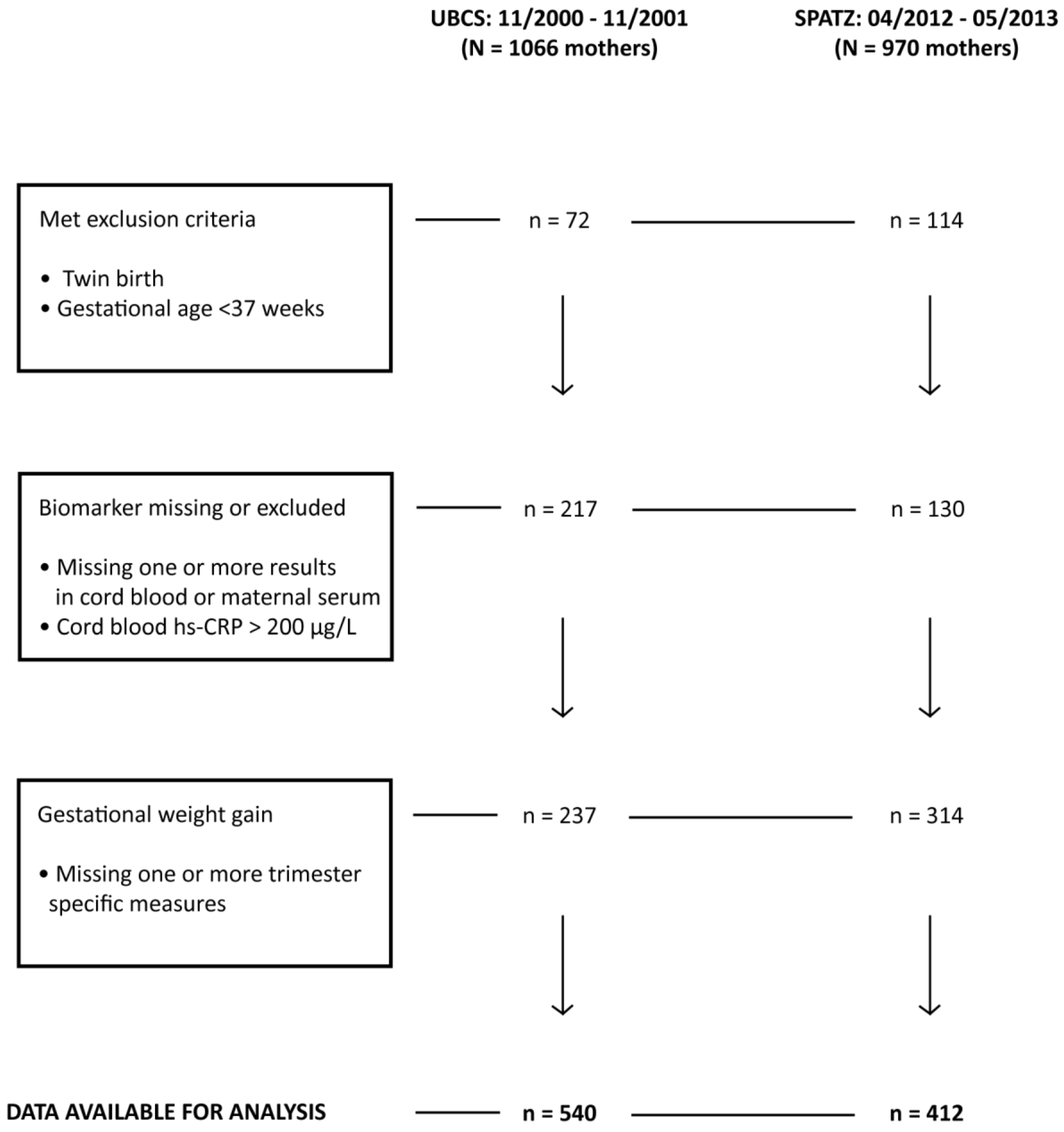

Supplement Table 1: Comparison of UBCS full cohort to final analysis subpopulation†

	Total Population (N = 1090)			Subpopulation (n = 589)		
**MATERNAL FACTORS**						
**Maternal age (years)**	1087	31.0	(30.7; 31.3)	539	31.0	(30.6; 31.4)
**Maternal education**						
≥12 years education	398	36.5%	(33.7%; 39.4%)	192	35.6%	(31.5%; 39.6%)
<12 years education	662	60.7%	(57.8%; 63.6%)	337	62.4%	(58.3%; 66.5%)
Missing	30	2.8%	(1.8%; 3.7%)	11	2.0%	(0.8%; 3.2%)
**Parity**						
First parity	541	49.6%	(46.7%; 52.6%)	276	51.1%	(46.9%; 55.3%)
Second or higher	540	49.5%	(46.6%; 52.5%)	260	48.1%	(43.9%; 52.4%)
Missing	9	0.8%	(0.3%; 1.4%)	4	0.7%	(0.0%; 1.5%)
**Smoking history (within 1 year before delivery)**						
No	740	67.9%	(65.1%; 70.7%)	361	66.9%	(62.9%; 70.8%)
Yes	347	31.8%	(29.1%; 34.6%)	179	33.1%	(29.2%; 37.1%)
Missing	3	0.3%	(0.0%; 0.6%)		.	
**Maternal pre-pregnancy BMI category**						
Underweight (BMI <18.5)	39	3.6%	(2.5%; 4.7%)	17	3.1%	(1.7%; 4.6%)
Normal (18.5 ≤BMI <25.0)	725	66.5%	(63.7%; 69.3%)	364	67.4%	(63.5%; 71.4%)
Overweight (25.0 ≤BMI <30.0)	227	20.8%	(18.4%; 23.2%)	120	22.2%	(18.7%; 25.7%)
Obese (BMI ≥30.0)	86	7.9%	(6.3%; 9.5%)	38	7.0%	(4.9%; 9.2%)
Missing	13	1.2%	(0.5%; 1.8%)	1	0.2%	(0.0%; 0.5%)
**PREGNANCY AND BIRTH**						
**Gender**						
Male	551	50.6%	(47.6%; 53.5%)	280	51.9%	(47.6%; 56.1%)
Female	537	49.3%	(46.3%; 52.2%)	260	48.1%	(43.9%; 52.4%)
Missing	2	0.2%	(0.0%; 0.4%)		.	
**Birth weight (g)**	1084	3379	(3351; 3407)	540	3447	(3410; 3484)
**Delivery mode**						
Vaginal spontaneous	854	78.3%	(75.9%; 80.8%)	448	83.0%	(79.8%; 86.1%)
Elective cesarean	65	6.0%	(4.6%; 7.4%)	23	4.3%	(2.6%; 6.0%)
Emergency cesarean	126	11.6%	(9.7%; 13.5%)	48	8.9%	(6.5%; 11.3%)
Vaginal assisted	45	4.1%	(2.9%; 5.3%)	21	3.9%	(2.3%; 5.5%)
**Duration of labor (hours)**	1049	8.4	(8.0; 8.7)	533	8.5	(8.0; 9.0)
**GESTATIONAL AGE AT GESTATIONAL WEIGHT MEASURE (days)**						
Beginning of trimester 2	930	84	(83.5; 84)	540	84.6	(84; 85)
Beginning of trimester 3	1000	190	(190; 191)	540	190	(190; 191)
Last measure	927	271	(270; 272)	540	274	(273; 275)
**GESTATATIONAL WEIGHT GAIN (kg)**						
Trimester 1	930	1.9	(1.8; 2.1)	540	2.0	(1.8; 2.3)
Trimester 2	873	7.4	(7.2; 7.6)	540	7.4	(7.2; 7.6)
Trimester 3	864	5.8	(5.6; 6.0)	540	5.9	(5.7; 6.1)
Total	921	14.9	(14.5; 15.2)	540	15.3	(14.9; 15.8)
**WEIGHT GAIN CATEGORY (IOM, 2009)**						
Low	177	16.2%	(14.0%; 18.4%)	85	15.7%	(12.7%; 18.8%)
Normal	294	27.0%	(24.3%; 29.6%)	175	32.4%	(28.5%; 36.4%)
Excessive	448	41.1%	(38.2%; 44.0%)	279	51.7%	(47.5%; 55.9%)
Missing	171	15.7%	(13.5%; 17.8%)	1	0.2%	(0.0%; 0.5%)
**CORD BLOOD MARKERS**						

Adiponectin (mg/l)	1008	31.7	(30.9; 32.6)	540	32.6	(31.4; 33.8)
Leptin (µg/l)	1007	10.9	(10.1; 11.7)	540	11.1	(10.1; 12.2)
hs-CRP (µg/l)	974	129.9	(43.6; 216.2)	540	50.1	(47.2; 52.9)
**POST-DELIVERY MATERNAL SERUM MARKERS**						
Adiponectin (mg/l)	1016	9.0	(8.7; 9.3)	540	9.3	(8.9; 9.7)
Leptin (µg/l)	1013	20.0	(18.6; 21.3)	540	21.1	(19.3; 22.8)
hs-CRP (mg/l)	993	72.1	(68.2; 75.9)	540	69.7	(64.5; 74.8)

† Statistics expressed as n and column percentage for categorical variables or median (25th percentile; 75th percentile) for continuous variables
* Column values may not always add up to total due to missing values for some variables
** p-value calculated as either χ^2or fisher's exact for categorical variables or Kruskal-Wallis for continuous variables

Supplement Table 2: Comparison of SPATZ full cohort to final analysis subpopulation†

	Total Population (N = 1006)			Subpopulation (n = 412)		
**MATERNAL FACTORS**						
**Maternal age (years)**	1006	32.7	(32.4; 33.0)	412	32.8	(32.4; 33.3)
**Maternal education**						
≥12 years education	577	57.4%	(54.3%; 60.4%)	250	60.7%	(56.0%; 65.4%)
<12 years education	410	40.8%	(37.7%; 43.8%)	155	37.6%	(32.9%; 42.3%)
Missing	19	1.9%	(1.0%; 2.7%)	7	1.7%	(0.5%; 2.9%)
**Parity**						
First parity	547	54.4%	(51.3%; 57.5%)	227	55.1%	(50.3%; 59.9%)
Second or higher	458	45.5%	(42.4%; 48.6%)	185	44.9%	(40.1%; 49.7%)
Missing	1	0.1%	(0.0%; 0.3%)		.	
**Smoking history (within 1 year before delivery)**						
No	730	72.6%	(69.8%; 75.3%)	307	74.5%	(70.3%; 78.7%)
Yes	265	26.3%	(23.6%; 29.1%)	100	24.3%	(20.1%; 28.4%)
Missing	11	1.1%	(0.5%; 1.7%)	5	1.2%	(0.2%; 2.3%)
**Maternal pre-pregnancy BMI category**						
Underweight (BMI <18.5)	27	2.7%	(1.7%; 3.7%)	12	2.9%	(1.3%; 4.5%)
Normal (18.5 ≤BMI <25.0)	599	59.5%	(56.5%; 62.6%)	257	62.4%	(57.7%; 67.1%)
Overweight (25.0 ≤BMI <30.0)	225	22.4%	(19.8%; 24.9%)	91	22.1%	(18.1%; 26.1%)
Obese (BMI ≥30.0)	124	12.3%	(10.3%; 14.4%)	50	12.1%	(9.0%; 15.3%)
Missing	31	3.1%	(2.0%; 4.1%)	2	0.5%	(0.0%; 1.2%)
**PREGNANCY AND BIRTH**						
**Gender**						
Male	523	52.0%	(48.9%; 55.1%)	226	54.9%	(50.0%; 59.7%)
Female	483	48.0%	(44.9%; 51.1%)	186	45.1%	(40.3%; 50.0%)
**Birth weight (g)**	1005	3278	(3245; 3311)	412	3438	(3395; 3481)
**Delivery mode**						
Vaginal spontaneous	639	63.5%	(60.5%; 66.5%)	274	66.5%	(61.9%; 71.1%)
Elective cesarean	125	12.4%	(10.4%; 14.5%)	53	12.9%	(9.6%; 16.1%)
Emergency cesarean	156	15.5%	(13.3%; 17.7%)	42	10.2%	(7.3%; 13.1%)
Vaginal assisted	85	8.4%	(6.7%; 10.2%)	43	10.4%	(7.5%; 13.4%)
Missing	1	0.1%	(0.0%; 0.3%)		.	
**Duration of labor (hours)**	952	7.6	(7.2; 8.0)	400	7.9	(7.3; 8.5)
**GESTATIONAL AGE AT GESTATIONAL WEIGHT MEASURE (days)**						
Beginning of trimester 2	906	86	(85; 88)	412	84	(84; 85)
Beginning of trimester 3	906	191	(190; 192)	412	190	(189; 190)
Last measure	906	268	(267; 269)	412	273	(272; 273)
**GESTATATIONAL WEIGHT GAIN (kg)**						
Trimester 1	597	1.8	(1.7; 2.0)	412	1.9	(1.7; 2.2)
Trimester 2	788	7.1	(6.9; 7.3)	412	7.0	(6.8; 7.3)
Trimester 3	776	5.5	(5.3; 5.7)	412	5.7	(5.4; 6.0)
Total	536	14.5	(14.0; 15.0)	412	14.7	(14.2; 15.3)
**WEIGHT GAIN CATEGORY (IOM, 2009)**						
Low	87	8.6%	(6.9%; 10.4%)	66	16.0%	(12.5%; 19.6%)
Normal	190	18.9%	(16.5%; 21.3%)	145	35.2%	(30.6%; 39.8%)
Excessive	256	25.4%	(22.8%; 28.1%)	199	48.3%	(43.5%; 53.1%)
Missing	473	47.0%	(43.9%; 50.1%)	2	0.5%	(0.0%; 1.2%)

**CORD BLOOD MARKERS**						
Adiponectin (mg/l)	891	30.5	(29.7; 31.3)	412	31.3	(30.1; 32.5)
Leptin (µg/l)	891	10.3	(8.6; 11.9)	412	10.0	(9.2; 10.8)
hs-CRP (µg/l)	891	53.9	(46.1; 61.6)	412	37.5	(35.1; 39.9)
**POST-DELIVERY MATERNAL SERUM MARKERS**						
Adiponectin (mg/l)	956	6.1	(5.9; 6.3)	412	6.3	(6.0; 6.6)
Leptin (µg/l)	956	18.3	(17.2; 19.3)	412	18.7	(17.1; 20.3)
hs-CRP (mg/l)	954	258.1	(20.1; 496.1)	412	81.0	(74.8; 87.1)

† Statistics expressed as n and column percentage for categorical variables or median (25th percentile; 75th percentile) for continuous variables
* Column values may not always add up to total due to missing values for some variables
** p-value calculated as either χ^2or fisher's exact for categorical variables or Kruskal-Wallis for continuous variables

Supplement table 3: Spearman correlation coefficients cord and maternal serum biomarkers and birth weight

		Adiponectin (maternal serum)	Leptin (maternal serum)	hs-CRP (maternal serum)	Birth weight
**Adiponectin (cord blood)**	UBCS	0.11**	0.11*	-0.03	0.09*
	SPATZ	0.18***	0.00	0.05	0.10*
**Leptin (cord blood)**	UBCS	-0.08	0.11*	0.11**	0.39***
	SPATZ	-0.20***	0.22***	0.13**	0.47***
**hs-CRP (cord blood)**	UBCS	-0.07	0.11**	0.34***	0.05
	SPATZ	-0.19***	0.25***	0.33***	0.03
**Birth weight**	UBCS	-0.06	-0.06	0.02	1.00
	SPATZ	-0.03	-0.01	0.08	1.00

UBCS subjects were recruited from 11/2000-11/2001; SPATZ subjects were recruited from 04/2012-05/2013

[*]Asterisks indicate p-value significance (*) < 0.05; (**) < 0.01; (***) < 0.001
